# Pre-fractionated Microbial Samples – The Second Generation Natural Products Library at Wyeth

**DOI:** 10.3390/molecules13061406

**Published:** 2008-06-20

**Authors:** Melissa M. Wagenaar

**Affiliations:** Natural Products Discovery Research, Chemical and Screening Sciences, Wyeth Research, 401 N. Middletown Road, Pearl River, New York 10965, USA; E-mail: wagenam@wyeth.com

**Keywords:** Natural products, pre-fractionated libraries, high-throughput screening, drug discovery, pharmaceutical industry

## Abstract

From the beginning of the antibiotic era in the 1940s to the present, Wyeth has sustained an active research program in the area of natural products discovery. This program has continually evolved through the years in order to best align with the “current” drug discovery paradigm in the pharmaceutical industry. The introduction of high-throughput screening and the miniaturization of assays have created a need to optimize natural product samples to better suit these new technologies. Furthermore, natural product programs are faced with an ever shortening time period from hit detection to lead characterization. To address these issues, Wyeth has created a pre-fractionated natural products library using reversed-phase HPLC to complement their existing library of crude extracts. The details of the pre-fractionated library and a cost-benefit analysis will be presented in this review.

## Introduction

Mankind has continually looked to Nature for new therapeutics to aid in the treatment of human diseases. From the early use of traditional medicines and natural poisons to the present day analytical investigations of plants and microbes by pharmaceutical companies for drug discovery, Nature has provided sources of and inspirations for novel drugs. Compounds of natural product origin including original natural products, compounds derived semisynthetically from natural products, and synthetic compounds based on natural product pharmacophores have proven particularly useful in the areas of oncology and anti-infectives. Approximately 60% of all available small molecule anticancer drugs from the 1940s to the present are of natural product origin. More impressively, compounds produced or inspired by natural products accounted for 75% of the 98 small molecule antibacterial New Chemical Entities (NCEs) from January 1981 to June 2006 [1]. These results are not surprising given that natural products have been selected through evolutionary pressures to interact with a variety of macromolecular targets with high binding affinity and selectivity.

Although natural products historically have been a significant source of therapeutic agents, many major pharmaceutical companies have reduced or eliminated their efforts in the area of natural products over the past twenty years. Several factors have contributed to this declining interest in natural products as a source for drug discovery. First, natural products research is viewed as time and resource intensive. The development of high-throughput screening (HTS) has enabled very efficient assay turnaround of a large number of compounds, thereby, reducing project cycle times. Second, the early promises of combinatorial chemistry to enable rapid production of large chemically diverse, “drug-like” libraries ideal for HTS also influenced the decision to downsize natural products drug discovery programs. Additionally, many major pharmaceutical companies have deemphasized research in the area of infectious disease, a traditionally strong area for natural products. Although difficult to ascertain and measure, emerging evidence suggests that the coupling of HTS and high-throughput organic synthesis has not matched the original expectations of supplying high value leads which in turn deliver NCEs to the market. Rather while R&D spending has increased over the past twenty years, the number of NCEs launched has actually declined. Newman and Cragg note that only one *de novo* NCE (sorafenib) resulting from combinatorial chemistry has been reported in the public domain to date and that the number of NCEs hit a 24-year low in 2005 [1]. Researchers at Pfizer propose that the output from HTS is dependent on the interrelationships between the quality of the compound library, the target and the screening process [2].

In an effort to capitalize on the success of natural products in drug discovery, several investigators have attempted to understand the relationships between natural products, marketed drugs and synthetically-prepared small molecule libraries. These studies have focused on the differences in structural properties of the three classes of compounds comparing molecular descriptors including but not limited to molecular weight, number of heteroatoms, and types of pharmacophore groups. When comparing structural similarities, Henkel *et al.* concluded that about 40% of the natural product structures studied are not represented in the pool of test synthetic compounds used [3]. These results were supported by Grabowski and Schneider, who identified more than one thousand scaffolds in the natural product library that were not exemplified in any of the other sets of compounds studied [4]. Feher and Schmidt used principal components analysis (PCA) to map the chemical diversity space of the three classes of compounds and found that, in general, trade drugs and natural products cover a much larger volume of the diversity space than do combinatorial compounds, and thus combinatorial compound libraries are much less diverse than those of natural products [5]. Similar results were described by Wetzel *et al.* for their PCA of sets of natural products, bioactive molecules and synthetic compounds with the exception that the overlap between drugs and natural products was found to be much smaller in their study [6]. Some of the features that distinguish natural products from synthetic small molecule libraries are the number of nitrogen and oxygen atoms, the number of chiral centers, and the ratio of aromatic atoms to ring atoms per molecule. Because most pharmaceutical companies are no longer actively involved in natural products discovery, researchers are now challenged with how to incorporate these natural product-like features into synthetically-prepared small molecule libraries so that these synthetic compounds reside within the same chemical space defined by trade drugs and natural products. Both Morphochem (now Biovertis AG) and Infinity Pharmaceuticals have used synthetic strategies to create compound collections with the goal of mimicking the features specific to natural products.

As a result of the unique structural properties of natural products and their exquisite biological activity, Wyeth has maintained an active research program in the area of natural products since the 1940s. Wyeth’s most recent natural products success is an anti-infective agent based on the tetracycline core; Tygacil^®^ was approved in 2005 for complicated skin and skin structure infections caused by designated microorganisms. Two other natural product-based drugs currently marketed by Wyeth are Rapamune^®^ and Mylotarg^®^. Rapamune^®^ is an immunosuppressant agent used in kidney transplant patients to prevent organ rejection whereas Mylotarg^®^ is indicated for the treatment of bone marrow cancer in a specific patient population. In order to remain a productive and essential component of Wyeth drug discovery and development, the natural products program has continually evolved through the years in order to assimilate into the “current” discovery paradigm in the pharmaceutical industry.

Drug discovery in today’s pharmaceutical environment is driven by high-throughput screening of large chemical libraries often exceeding 1 million compounds and ever shortening project cycle times. Target-based screens and particular technology platforms are best suited for the HTS paradigm. Often the screens developed for use in HTS are designed for screening synthetically-prepared libraries of pure compounds and not crude microbial extracts containing tens to hundreds of compounds. Furthermore, the desire to screen enormous compound collections has resulted in the miniaturization of every component of an assay. Today’s assays are typically run in 384 or 1536 well plate formats corresponding to total assay volumes between 2 and 20 μL per well with dispensation in the submicroliter range. This new age of drug discovery characterized by HTS, a rapid hit-to-lead phase, and miniaturization creates several challenges for the area of natural products research.

Approximately 15 years ago, when the concept of HTS was first embraced by the pharmaceutical industry, Wyeth began creation of a library of crude natural product extracts for testing in HTS as did many other major pharmaceutical companies. Crude extract libraries have several advantages: inexpensive to prepare; minimal sample preparation time; moderate overall size; high degree of diversity. However, after several years of screening these libraries, it is now well recognized that these advantages may be easily outweighed by the commonly reported limitations of crude extract libraries including: 1) the physical nature of the samples makes them unsuitable for automated liquid handling systems (too viscous); 2) minor metabolites may go undetected in crude extracts or be masked by other components in the complex mixture; 3) time- and resource-intensive follow-up is needed to isolate and identify active components; 4) known structures are often re-discovered; and 5) chemically unattractive compounds are frequently isolated. As a result of these limitations, a second generation natural products library of pre-fractionated extracts was prepared.

Based on the knowledge acquired over 10 years of screening crude extracts, it was evident that the design and production of the natural products library is of significant importance. Pure compound libraries of natural products appear to address many of the disadvantages related to crude libraries and could represent the ideal solution for natural products research in the current drug discovery high-throughput paradigm. As with synthetically-prepared small molecule libraries, a library of pure natural products should be strategically designed to provide representative coverage of the desired chemical space. However, the time- and resource-intensive steps have not been eliminated with this type of library design but simply shifted to pre-HTS. Additionally, more sophisticated infrastructure and cheminformatics are needed upfront to assess the potential of extracts for compound production and to aid in the elimination of redundant and ubiquitous compounds. Furthermore, minor components may continue to go undetected depending on the peak detection and isolation methods utilized in preparing the pure compound library and thus would not be included in the screening deck. While some common natural products are readily available from commercial sources at a reasonable price, the majority would need to be isolated in-house or obtained through partnerships. The costs associated with isolation and characterization of individual compounds can be prohibitive and the isolation of > 1 mg quantities of many compounds is time- and resource-intensive [7]. Therefore, the overall chemical diversity of a pure compound natural products library is limited by the availability of desirable compounds and the prohibitive costs associated with the isolation of targeted metabolites.

In order to obtain a moderate degree of chemical diversity while addressing some of the issues inherent to crude extracts, a pre-fractionated natural product library was prepared for use in the high-throughput screening paradigm at Wyeth. The simple definition of pre-fractionation is the fractionation of a crude extract prior to biological testing. Depending on the method(s) used for pre-fractionation, the number of compounds in the original crude extract, and the goals of the fractionation, the resulting fractions can vary widely in complexity from a mixture of multiple compounds to a single major compound of >90% purity. Several factors should be considered when planning a pre-fractionated library including the cost, time and resources needed for production, the cost per sample per bioassay, the desired number of compounds per fraction, and the degree of diversity desired for libraries of a fixed size. Most pre-fractionation strategies spread the cost and resources needed to effectively run a natural products program over the entire discovery process from pre-HTS sample preparation to post-HTS hit resolution.

Several companies have reported methods for generating pre-fractionated libraries including, but not limited to, Sequoia Sciences, Inc. [8], MerLion Pharmaceuticals Pte Ltd [9], Hans-Knoll Institute for Natural Products Research [10], bioLeads GmbH [11] and Unigen Pharmaceuticals, Inc [12]. Butler *et. al*. (MerLion Pharmaceuticals) report preparation of a basic pre-fractionated library using reversed-phase HPLC (RP-HPLC) with an acetonitrile/water gradient. The reported library of more than 120,000 fractions was generated by separating crude extracts into four fractions based on elution time and plating the family of fractions alongside the crude for screening. Analysis of the library indicated good overall resolution as determined by the presence of activity in only one of the four fractions for 84% of the actives and an overall increase in activity in the fractions when compared to the crude [9]. Sequoia Sciences, Inc. describes a more sophisticated, multi-step process for creating their fractionated library of plant extracts. After initial processing and extraction, each organic extract is subjected to silica gel flash chromatography resulting in four sub-fractions. These four sub-fractions are then further fractionated by RP-HPLC generating 40 fractions per sub-fraction for a total of 160 fractions per organic extract. An aqueous extract is also prepared and subjected to RP-HPLC to generate an additional 40 fractions resulting in a total of 200 fractions per plant sample. Analysis of the fractions indicated that 60% of the fractions contain “quantifiable” compounds as determined by LCMS-ELSD and the approximate number of compounds per fraction ranges between one and five [8]. 

Wyeth maintains a library of crude extracts generated from 1996 through 2002 representing more than 20,000 microbial organisms. To complement this original library and to provide higher quality samples, a pre-fractionated natural product library was prepared over a four-year period at Wyeth. This paper reviews the method used to create the pre-fractionated natural products library at Wyeth and some of the strategic decisions made while developing the process. Additionally, the benefits of the pre-fractionated library in the drug discovery pathway are described and evaluation of this library using HTS data is discussed.

## Results and Discussion

### Library Preparation

The overall strategic design of the library was influenced by three main factors: cost, quality and time. The goal at the outset was to achieve the highest quality library possible at a moderate cost and within a reasonable time with the size of the natural products library not exceeding more than 10% of the entire screening library. Financial considerations not only include the total cost of library preparation from organism isolation to fermentation to fractionation and plating but also the cost to screen each sample. At a cost of 25 to 50 cents per well per assay, the cost can rapidly escalate. In this instance, screening a library of one million wells would cost between $250K and $500K per assay. Additionally, features that enable reduction in project cycle time post-HTS were considered in the design of the library. Simplified mixtures should help to reduce the time from hit identification to compound characterization as should the immediate availability of stored crude extract.

**Figure 1:**
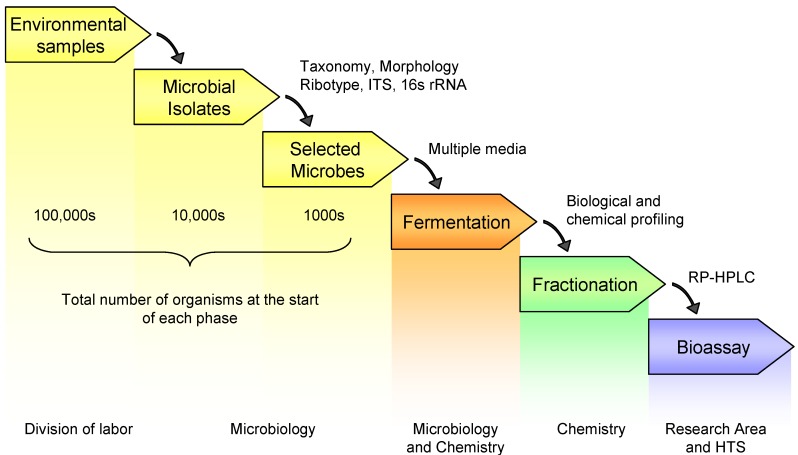
General schematic for library generation and screening.

Figure 1 outlines the workflow for the production and screening of a pre-fractionated library and the division of labor. This process represents a joint effort between microbiology, chemistry and HTS. The initial steps in library generation result in the selection of a set of microorganisms by microbiologists. Environmental samples are collected for isolation of microbes that are then selected based on various criteria for fermentation. These first three steps are not unique to the preparation of a pre-fractionated library. However, because of the increased workload in downstream processing and the need to generate high quality within a fixed library size, more rigorous selection criteria need to be applied in the selection of microbes. More time and resources will be needed upfront for the pre-screening of strains using molecular techniques as well as classical taxonomy to remove redundancy and select for novel organisms. The selected microorganisms are then fermented using at least two different fermentation conditions to increase the potential for producing chemical diversity. The liquid fermentations are pooled prior to solid-phase extraction. By pooling the fermentations of each individual organism prior to extraction, the overall cost and time for extraction, profiling and fractionation are reduced by fifty percent. The resulting crude extracts are subjected to both biological and chemical profiling. Chemical profiles of the extracts are generated by LCMS-DAD-ELSD and analyzed for the presence of secondary metabolites. This chemical screening aids in the dereplication of known compounds and thus can be used to reduce redundancy in the overall library. Biological profiling in this instance involves the testing of the extracts in antimicrobial assays; the presence of antimicrobial activity is one indication that an organism is producing secondary metabolites. Biological screening can be more sensitive than chemical profiling and is useful for detecting minor compounds present in quantities below the limit of detection of the LCMS-DAD-ELSD. Select extracts are then fractionated and the fractionated samples plated and sent to the research areas and/or the HTS group for testing. Based on analysis of over 1000 combined extracts, it was determined that approximately 65% of the organisms fermented using our standard fermentation conditions produced secondary metabolites and were suitable for fractionation. Because only limited chemical and biological data is acquired on each sample, the organic extracts that are not fractionated are incorporated into the library as unfractionated crude extracts.

Extracts meeting the criteria for fractionation are concentrated and divided into two equal portions. One portion, the stored crude, is concentrated to dryness and stored at –20 ºC as reference material for processing should that sample show activity later when tested. The remaining half is used for fractionation and plating. Basic separation can be achieved with a number of different techniques such as solid-phase extraction, liquid-liquid partitioning, and column chromatography. Preparative reversed-phase HPLC was chosen as the method for fractionation because it is robust and reproducible, can be easily automated, requires minimal sample handling, and provides good resolution and recovery.

**Figure 2:**
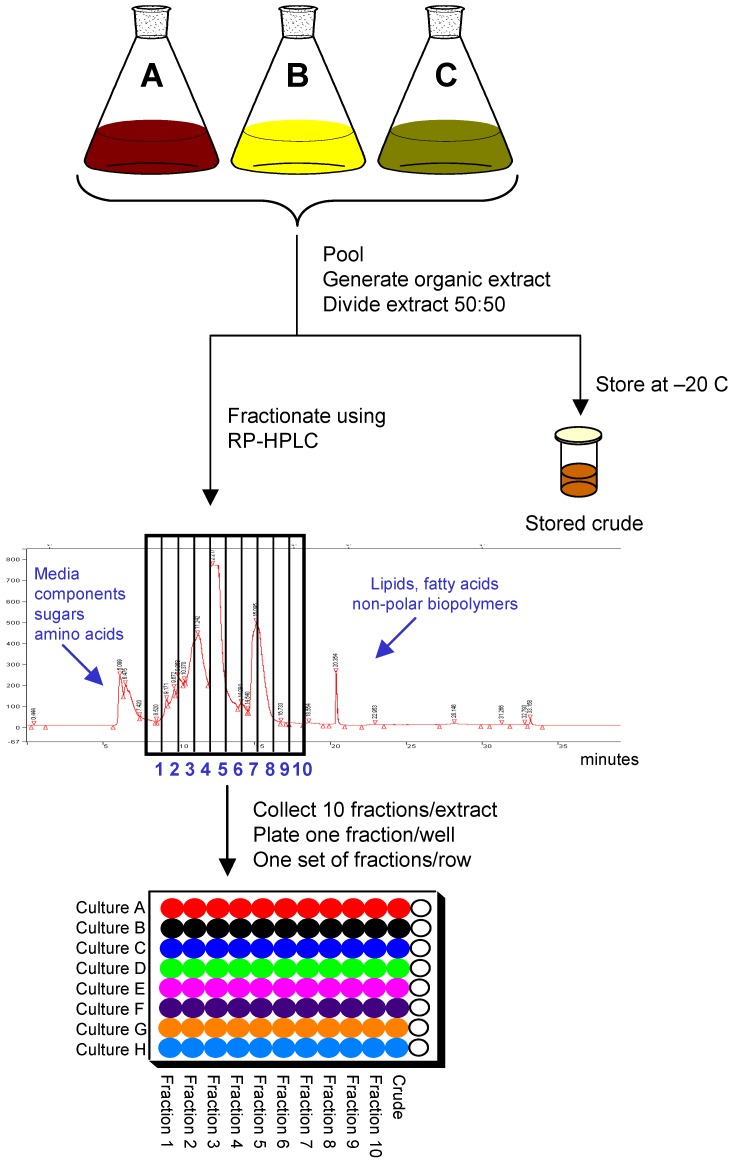
Detailed overview of the production process for the pre-fractionated natural products library.

In order to balance the degree of resolution with the biological and chemical diversity while working within the library size dedicated for natural products, a total of ten fractions were collected per extract. Figure 2 provides an overview of the fractionation scheme. To prepare for fractionation, the second half of the sample is resolubilized and one-tenth of that solution is removed for plating as the crude in column 11 of the 96-well plate used by the HTS group. Thus the total “saved” crude material plated for HTS is equivalent to 1/20^th^ of the original fermentation volume. The remaining nine-tenths of that solution is subjected to RP-HPLC using a Luna C-18 column to generate 10 fractions collected in 1-minute increments. Because the intent is to collect the fractions most likely to contain compounds with drug-like properties, the early eluting material consisting of media components and highly polar compounds is not collected nor is the late-eluting lipophilic portion. The total run time for each sample including a wash cycle of the column is 35 minutes. In an average 8-hour workday a total of thirteen runs can be completed. With a fully automated system, higher capacity can be achieved. The rate-limiting step in the fractionation is the drying of the fractions. Each SpeedVac system can handle two sets of fractions in a 24-hour period.

Dried fractions are resuspended in DMSO as a 100X concentrate. The 1/10 “saved” crude is resuspended in an equal volume of DMSO; thus the crude is roughly 10 times less concentrated than the fractions. Samples are then dispensed into 96-well plates using a Tecan robotic system. The resulting 96-well plate consists of samples from eight different cultures with the column number corresponding to the fraction number, column 11 containing crude extract, and column 12 empty and available for controls.

### Library Analysis

**Figure 3:**
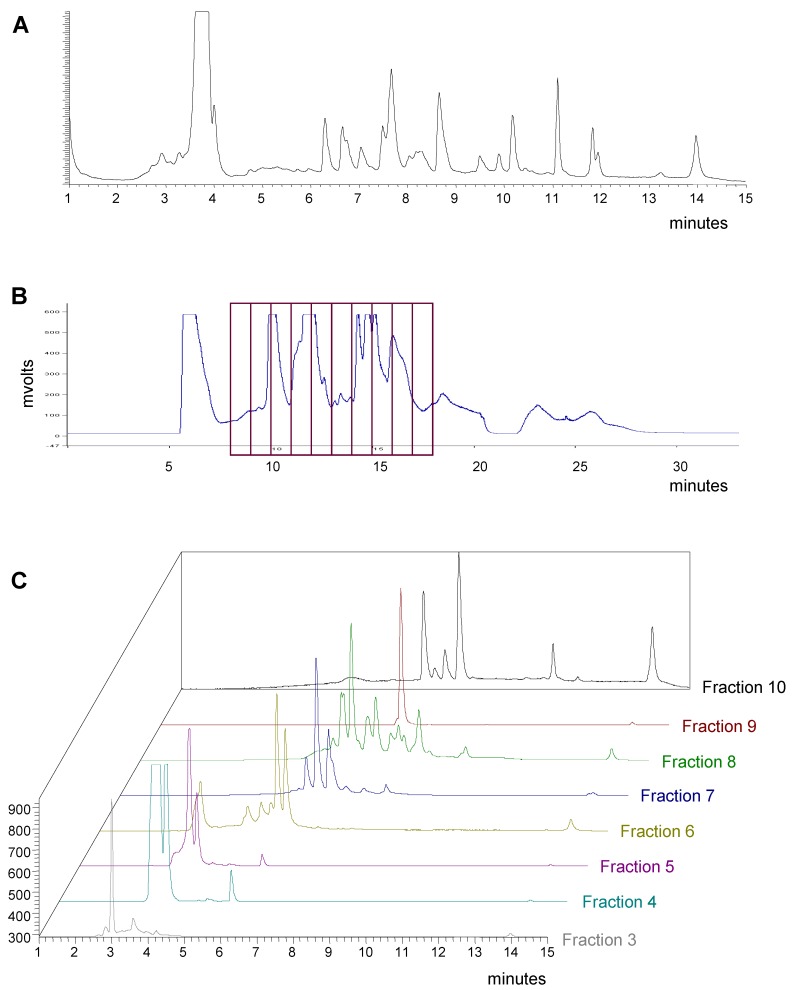
HPLC chromatograms of fungal extract 70360-F11. A) Analytical HPLC-ELSD chromatogram of the crude methanol extract 70360-F11. B) Preparative LC chromatogram of the crude methanol extract 70360-F11 (UV detection at 254 nm) with the fraction collection outlined. C) HPLC-ELSD chromatograms of fractions 3 through 10 from the preparative LC run of 70360-F11 shown in B.

Based on simple visual examination of chromatograms, the pre-fractionation method shows several qualitative benefits. Early-eluting media and polar components are removed from the samples. The overall complexity of the samples to be tested in HTS is reduced with fewer compounds per well. Suitable resolution was achieved and the major components that may interfere or mask the activity of minor components are separated into individual fractions. These benefits are illustrated in the example shown in Figure 3. Crude extract 70360-F11 contains more than 20 compounds as shown in the analytical HPLC-ELSD chromatogram (Figure 3A). Based on the biological and chemical profiling results, this extract was chosen for preparative fractionation. The fractions were collected in 1-minute intervals beginning at 8 minutes as shown on the HPLC-UV chromatogram (Figure 3B). The most polar components eluting between 0 and 7 minutes were discarded. The analytical HPLC-ELSD chromatograms of fractions 3 through 10 are shown in Figure 3C. Each fraction shows reduced complexity compared to the original crude extract with fraction 9 appearing to contain a single component. In addition, the majority of the sample by weight is separated into fraction 4 as determined by light scattering detection.

**Figure 4:**
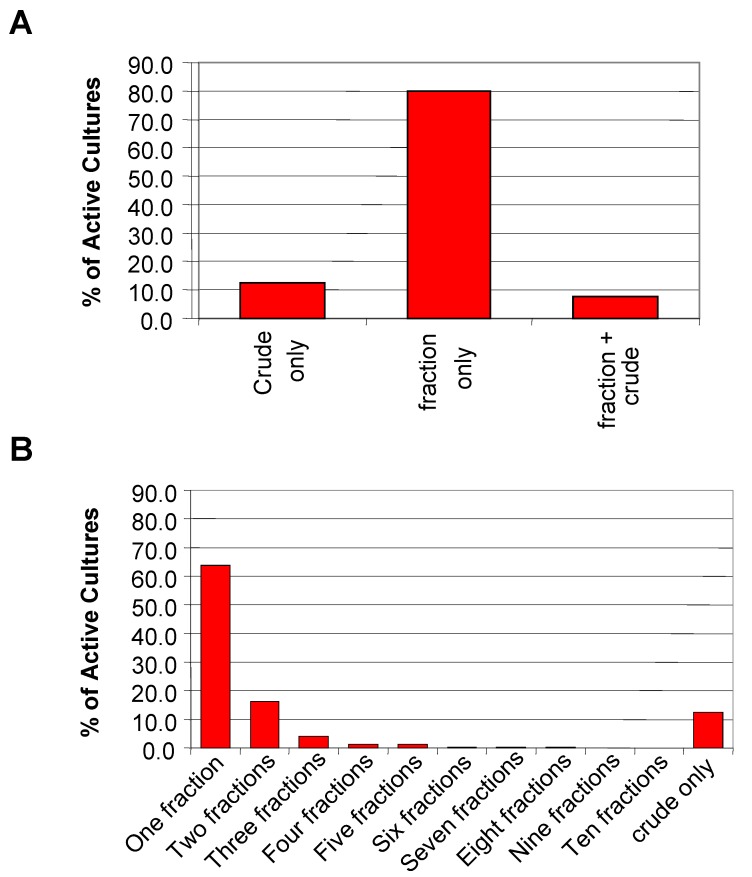
Distribution of the hits from nine HTS screens. A) General categorization of the HTS hits based on activity localization for 1882 active cultures. B) Detailed view of the activity distribution of the hits for the 1882 active cultures.

High-throughput screening data was used to do a more rigorous quantitative analysis of the pre-fractionation method. A total of 2750 primary hits from nine HTS assays were analyzed. The 2750 hits represent fractions and crude extracts from 1882 unique organisms. In 79.9% of the 1882 active cultures, the activity was found only in the fractions (Figure 4A). These samples would have been missed if only the crude extracts had been screened. It is unclear from the analysis if this finding is a result of the increase in concentration of the fractions compared to the crude or the removal of interfering compounds. However, it should be noted that the increase in concentration was only achievable because the physical nature of the samples (i.e. viscosity) was improved by pre-fractionation.

For 12.5% of the active cultures, the activity was detected only in the crude fraction reinforcing the decision to plate the crude sample alongside its fractions. This result suggests that the active compound eluted outside the time range for collection, was unstable under the conditions used for fractionation, or was retained on the column. For more than 60% of the active cultures, the activity was concentrated into a single fraction with an additional 16.2% of the active cultures registering activity in only two of the ten fractions (Figure 4B). Less than 1% of the active cultures showed activity spread over six or more fractions. Thus sufficient resolution was achieved with the pre-fractionation RP-HPLC method and collection parameters used.

**Figure 5:**
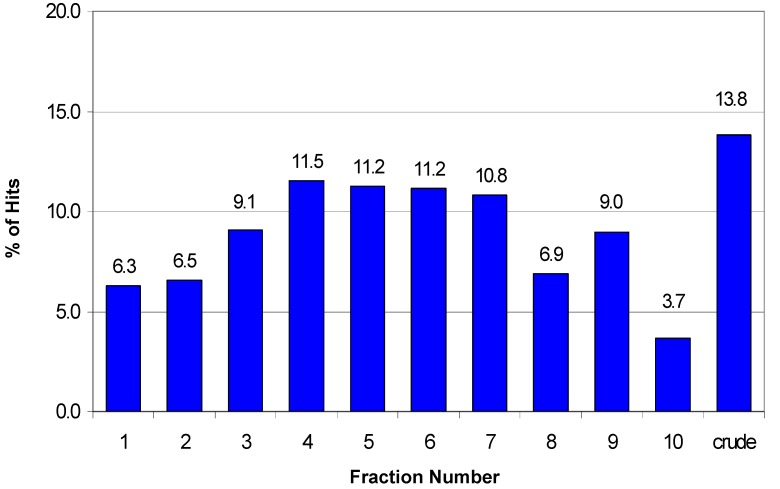
Distribution of the hits across fractions for nine HTS assays (total number of hits = 2750).

**Figure 6:**
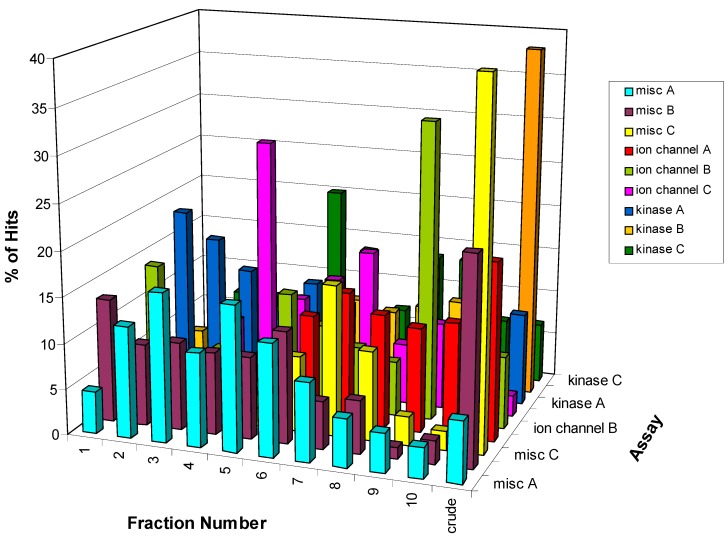
Distribution of the 2750 HTS hits from 9 screens showing the assay-dependency.

Figure 5 depicts the distribution of hits across the fractions for the 2750 hits identified in nine HTS assays. The pattern of activity across fractions approximates a Gaussian curve centered around fractions 5 and 6 with slightly fewer active samples found in the peripheral fractions. However, this distribution is clearly assay-dependent as can be seen when the activity pattern for each individual assay is analyzed as shown in Figure 6. For three of the nine assays the crude fraction represents the highest percentage (>20%) of the hits in those specific assays (misc B, misc C, and kinase B). However, the greatest number of hits is found in a single fraction for some of the other assays. For example, 33% of the hits from the ion channel B assay are found in fraction 9 while fraction 4 represents 28% of the hits in the ion channel C assay. In some instances, the HTS hits are distributed over all 10 fractions plus the crude with no one fraction corresponding to >20% of the hits. The distribution of hits over all ten fractions suggests that any reduction in the collection interval would result in missed actives. Furthermore, these data could help in the analysis of the hits and the prioritization of the hits for follow-up as described below.

**Figure 7:**
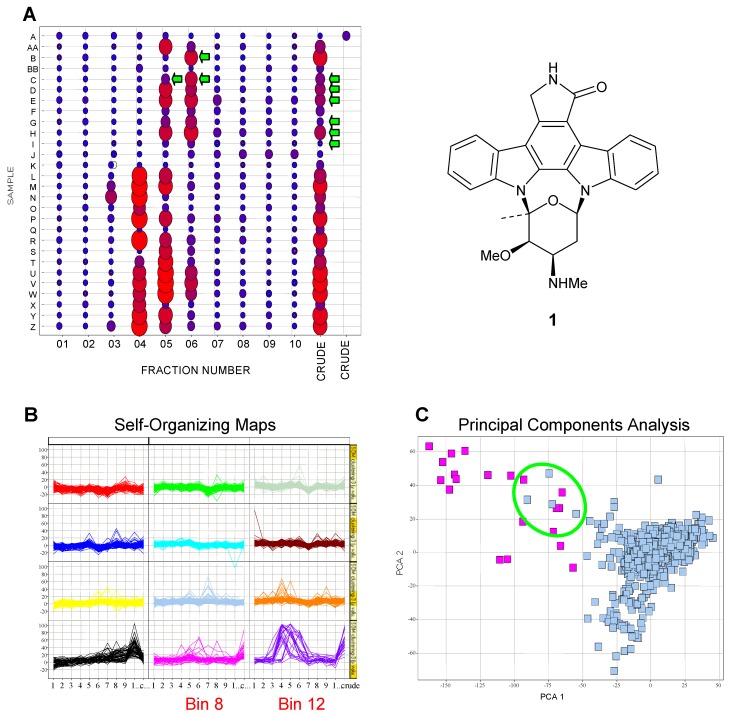
Examples of pattern recognition for actinomycete cultures tested in a kinase screen. A) Activity profiles for twenty-eight active actinomycete cultures. Color and size of the circle correlate with activity. Green arrows indicate samples known to contain staurosporine. B) Self-organizing map of the activity profiles for 1436 actinomycete cultures in a kinase screen. C) Principal components analysis of the activity profiles for 1436 actinomycete cultures. Samples in pink are known to contain staurosporine.

One of the major challenges in screening natural product crude samples is the prioritization of hits for follow-up. Even with screening a pre-fractionated library, the resolution of hits can be resource-intensive and thus focusing on the samples with the highest probability for success is preferential. Dereplication, the process of identifying known compounds, is possible with both crude and pre-fractionated extracts but there is no guarantee that the compound identified is responsible for the observed biological activity without additional experimental work. Differentiating between the hits that contain compounds of interest and those that contain ubiquitous or chemically unattractive compounds is not easily accomplished without proceeding through at least one round of bioassay-guided fractionation. A total of eleven data points are generated for each culture in the pre-fractionated library in comparison with a single data point per culture in the crude extract library. This additional information creates the opportunity for more effective data mining which can facilitate the selection of the best candidates for follow-up chemical processing and analysis. Comparison of the activity profiles of each culture can be informative. For example, the bioactivity profiles of 28 active actinomycete cultures tested in a kinase screen are shown in Figure 7A. The potency of each sample is represented both by the size and the color of the circle (larger circle = greater activity; red = most active). Based on information captured in our in-house database, several of the samples (fractions and crudes) as indicated with green arrows have previously been identified as containing staurosporine (**1**), a well-known kinase inhibitor [13]. The seven cultures known to contain staurosporine show similar activity profiles suggesting that staurosporine is responsible for the observed activity and is found in fractions 5 and 6. A similar pattern of activity is seen for 16 additional extracts (samples AA, F, L-P and R-Z). Analysis of the LCMS-DAD-ELSD chromatograms collected during the chemical profiling prior to fractionation indicated that at least 13 of the sixteen extracts with activity between fractions 4 and 6 contain staurosporine. Although fraction 4 is the active fraction of sample X, this sample does not appear to contain staurosporine by LCMS-DAD-ELSD analysis. This pattern recognition allowed for the rapid identification of the known compound staurosporine and the deprioritization of 20 of the initial active cultures. The observed shift in activity from fractions 5 and 6 to fractions 4 and 5 corresponds to a modification in the preparative HPLC fractionation method and collection parameters.

The visual inspection of bioactivity profiles is not suitable for the massive data sets produced by high-throughput screening. Pattern recognition tools including self-organizing maps and principal components analysis are available for triaging cumbersome data sets. More than 15,000 data points were used to generate activity profiles for each fractionated extract by plotting the fraction number versus the activity for 1436 actinomycete extracts. The activity profiles were then clustered using a self-organizing map algorithm in Spotfire^®^ and binned as shown in Figure 7B. Many of the extracts showed no activity and resulted in flat bioactivity profiles. Of the 25 extracts represented in bin 12, nineteen are known to contain staurosporine. The additional six cultures in bin 12 have a high probability of containing staurosporine. One additional staurosporine producer is located in bin 8. The bioactivity profiles can also be used in principal components analysis. In Figure 7C, the samples known to produce staurosporine are shown in pink and are separated from the majority of the other extracts shown in light blue. Several extracts (circled in green) are in close proximity or overlap with the staurosporine-containing cluster and thus should be evaluated further for the presence of staurosporine.

**Figure 8:**
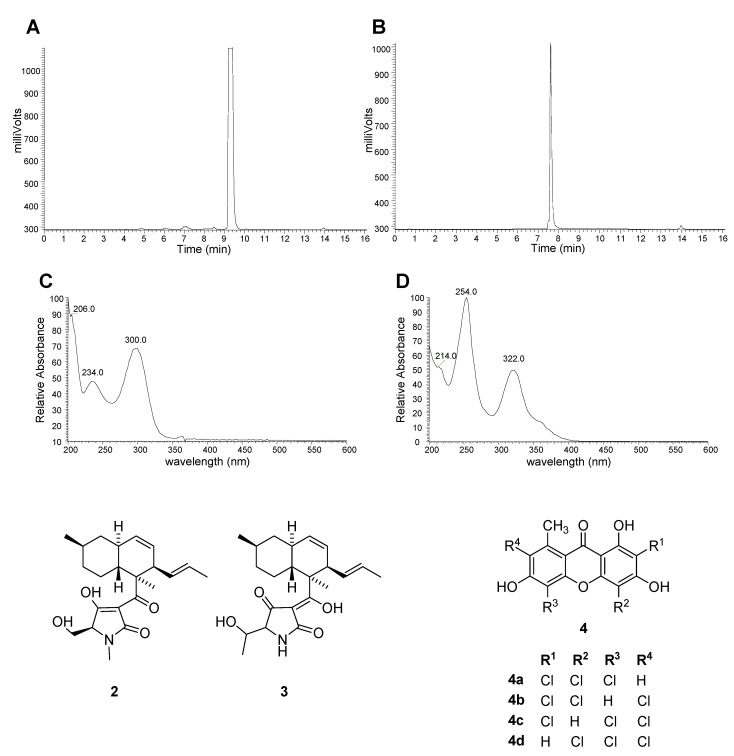
Analytical data for select samples containing one major component. A) HPLC-ELSD chromatogram of 70254-F08. B) HPLC-ELSD chromatogram of 70360-F09. C) UV spectrum of 70254-F08. D) UV spectrum of 70360-F09.

Another implication of the high-throughput drug discovery pathway is the shortened project cycle times. Screening of pre-fractionated natural product libraries reduces the number of bioassay-guided fractionation cycles needed to isolate and identify the active component(s) and thus the initial hits for a project are more rapidly resolved. As shown previously in Figure 3, the overall complexity of the pre-fractionated samples tested in HTS is greatly reduced which facilitates the isolation and identification process. In some instances, the samples generated by the pre-fractionation method contain one major component and no further isolation work may be needed as shown in the LCMS-ELSD chromatograms for two hits in a kinase assay (Figure 8A and Figure 8B).

High-resolution mass spectral (HRMS) data and UV spectra can be readily obtained for these samples and used for dereplicating the sample. A molecular formula of C_22_H_31_NO_4_ was determined using HRMS (*m/z* 374.23236 [M + H]^+^) for sample 70254-F08, a fraction from fermentation of a Fusarium sp. Combining the molecular formula with the UV spectrum (Figure 8C), sample 70254-F08 was identified as a tenuazonic acid derivative. The UV spectrum and molecular formula are consistent with both equisetin (**2**), a known antimicrobial metabolite produced by *Fusarium equiseti* [14], and the closely related paecilosetin (**3**) produced by *Paecilomyces farinosus* [15]. Similarly, the major component of 70360-F09 was determined to be a member of the norlichexanthone (**4**) family based on molecular formula (C_14_H_7_Cl_3_O_5_; *m/z* 358.92850 [M – H]^-^) and comparison of the UV spectrum (Figure 8D) with literature values for that of arthothelin (**4a**) [16, 17]. The dereplication and identification process can be greatly facilitated by the availability of a comprehensive UV spectral library.

**Figure 9:**
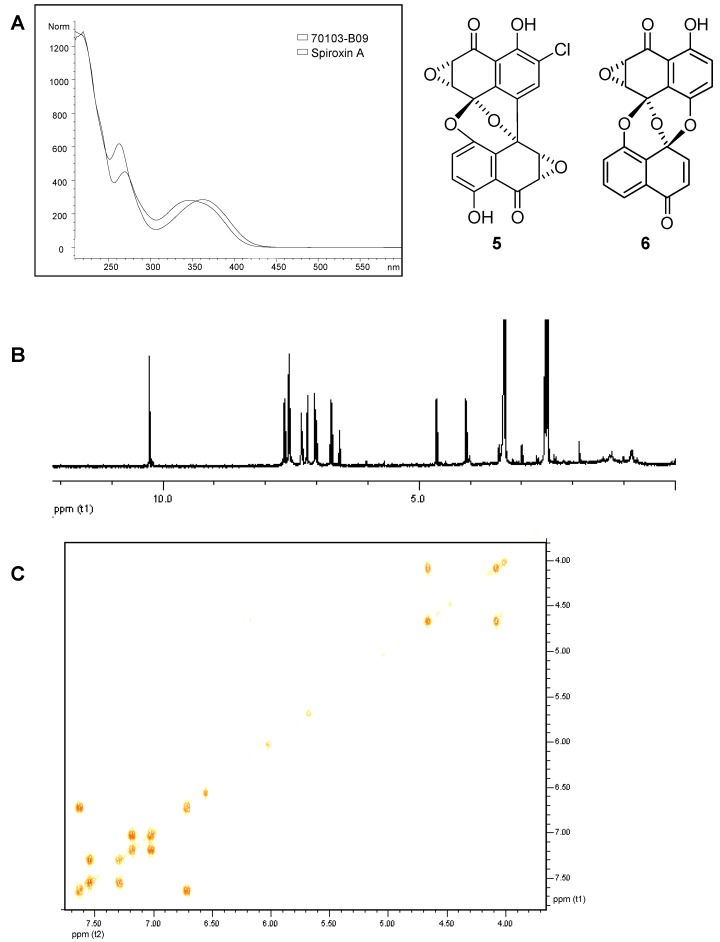
Analytical data for 70103-B09 used for identification of the major component. A) UV spectrum of 70103-B09 overlaid with the UV spectrum of spiroxin A (**5**). B) ^1^H- NMR spectrum of 70103-B09 in DMSO-d6. C) ^1^H-^1^H COSY spectrum of 70103-B09 in DMSO-d6.

Full structural characterization may be possible if enough sample is available. Sample 70103-B09 was an initial hit from high-throughput screening against a kinase target. Searching our in-house library of UV spectra, the UV spectrum of 70103-B09 showed similarity to that of spiroxin A (**5**) (Figure 9A) but it was not an exact match [18]. High-resolution mass spectral analysis indicated a molecular formula of C_20_H_12_O_7_ for the major component in sample 70103-B09 whereas spiroxin A has a molecular formula of C_20_H_9_ClO_8_. A 100 μL aliquot of the 100X solution of sample 70103-B09 was concentrated to dryness and resuspended in 200 μL of DMSO-*d*6 (concentration of major component is unknown). The ^1^H-NMR and COSY spectra of this fraction acquired using a Bruker 3 mm probe on a Bruker Avance 400 MHz spectrometer are shown in Figure 9B and Figure 9C, respectively. Based on a combination of the HRMS, the UV spectrum and the NMR spectral data, the major component in this fraction is consistent with preussomerin G (**6**). Preussomerin G was first isolated as an inhibitor of Ras farnesyl-protein transferase; this observed activity may be due to its ability to act as a Michael acceptor for strong nucleophiles [19]. Although preussomerin G is a known compound, this example illustrates that full structure elucidation may be possible for novel compounds if enough material is available to obtain all homo- and heteronuclear correlations by high-resolution NMR.

In instances when the active component cannot be readily dereplicated or fully characterized from the available DMSO stock solution, the stored crude can be used for follow-up of the HTS hit. Because project cycle times are shortened in the high-throughput paradigm and the refermentation phase is typically one of the lengthiest steps in the process from identifying an active extract to resolving the active constituent, the decision was made to create a stored crude during the library generation phase. This aspect of the library design is not specific to pre-fractionated libraries but can be applied to the production of any library type including crude extract libraries. Stored crude material offers two main benefits. First, in cases where the stored crude contains enough material for isolation and identification, the overall process is reduced by at least 4 to 6 weeks corresponding to the amount of time needed to revive the organism from the cryovial, ferment the culture, and then process the fermentation to generate a crude organic extract. Second, the stored crude is identical to the original crude sample tested in HTS and thus eliminates the need for confirmation of the bioactivity. Because refermentations involve living organisms, the activity is not always reproducible and a freshly prepared extract must be assayed to confirm that the original observed activity was reproduced during the refermentation. The average turnaround time is 1 to 2 weeks for confirmation of bioactivity. Therefore, the availability of stored crude material can reduce the overall time from hit identification to resolution by up to 8 weeks. The processing of the frozen stored crude can follow two distinct paths. The standard path forward involves bioassay-guided fractionation where the samples are tested for activity after each round of separation. Alternatively, in select cases, the active constituent can be pinpointed from the sample tested by HTS or by preliminary work with an aliquot of the DMSO stock solution but the amount of material obtained is insufficient for full structure elucidation. In these instances, the path forward employs target-guided isolation from the stored crude using UV or MS data in combination with retention time. This path reduces the time to resolution by eliminating the need for all bioassay support during the isolation process.

The pre-fractionated natural products library offers many strategic advantages over a library of crude extracts as described above. However, associated with these advantages are increased costs. Some costs can be easily measured while others are less tangible. First, because the samples are 10-fold more concentrated than the previously prepared crude extract library and a stored crude is generated, 20 times more organic extract needed to be produced. This increase in extract amount is directly proportional to an increase in raw materials such as the media for fermentation and the solvents for extraction and processing. Other additional costs include the initial investment in enabling technologies dedicated to library preparation including the various instrumentation needed for molecular analysis of the microorganisms, for profiling of the extracts, and for HPLC separation of the extracts. A regular supply of reagents associated with each technique also increased the overall production cost. One extra FTE per year and additional expertise was needed to support the library production. More difficult to ascertain is the cost associated with biological and chemical diversity. The overall throughput for generation of the pre-fractionated library was approximately 4-fold less than that of generating a library of crude extracts. This reduction in throughput translates to a moderate decrease in overall diversity. However, the pre-fractionation protocol results in a higher quality library with samples that are more suitable for the high-throughput drug discovery paradigm currently popular with the pharmaceutical industry. Because the ultimate goal was to increase the probability of success, the quality of the library and the resulting HTS hits is more important than the quantity of hits.

## Conclusions

While natural products have historically been a source of new drug leads, traditional natural products research is a labor- and cost-intensive endeavor incompatible with today’s drug discovery paradigm. High-throughput screening coupled with high-throughput organic synthesis has accelerated the pace of drug discovery and revolutionized the hit-to-lead process. However, success of this paradigm is co-dependent on the quality of the screening library and the screening process. To continue to play an integral role in the drug discovery program, a pre-fractionated extract library of microbial natural products was prepared to complement the existing crude extract library. A pre-fractionated natural products library represents a compromise between crude fermentation extracts and pure natural product compounds. The moderate diversity, intermediate size, and reduced sample complexity are balanced with the cost and time for production. Extracts were selected for fractionation based on limited biological and chemical profiling. On average, 65% of the organic extracts were determined to be suitable for fractionation based on this profiling. Each crude extract was fractionated into ten fractions and plated alongside the corresponding crude extract to create the pre-fractionated library for HTS.

This pre-fractionated library has demonstrated several advantages over the crude library in the path from hit identification to lead characterization. First, screening of the fractions results in a four-fold increase in the number of active cultures detected as compared to the crude extracts, thereby generating more leads for evaluation. Second, assay data for each culture consists of eleven data points per screen as compared with a single data point per crude extract per screen. Activity profiles created with these data can be used for triaging the hits so that redundant and undesirable compounds are eliminated and the most promising cultures are pursued. Furthermore, the pre-fractionated samples for screening are, in general, less complex and less viscous than crude extracts. The overall improvement in the physical state of the samples makes them more amenable to automated liquid sample handling in the microliter range used in HTS. Additionally, the reduced complexity aids in the dereplication process and can reduce the number of cycles of bioassay-guided fractionation needed to isolate and identify the active components. Finally, the availability of a stored crude created during the pre-fractionation process can significantly reduce the time needed to resolve the initial hits for a project.

The pre-fractionation protocol results in a higher quality library with samples that are more tailored for the high-throughput drug discovery paradigm. The quality of the library and the resulting HTS hits in conjunction with the increased possibilities for data mining and analytical sample investigation should translate into new opportunities to exploit the unique properties of natural products.

## Experimental

### General

Preparative HPLC separations were performed using a Luna 10μ Prep C18 (2) column (100 x 50 mm, 10 micron, 100A, Phenomenex, Inc., Torrance, CA) with a Luna 10 C18 (2) guard column (50 x 21.20 mm, 10 micron, 100A). Varian Star chromatography software was used to control the preparative HPLC consisting of two Dynamax SD-1 gradient HPLC pumps, a Varian ProStar 320 UV-Vis detector with a preparative flow cell, and a Varian model 701 fraction collector. Thermo Savant SpeedVac systems equipped with an SC210A concentration bowl and an RVT4101 refrigerated solvent trap were used to concentrate the preparative HPLC fractions to dryness. A Tecan Genesis RSP 200 was used for liquid handling to transfer the HPLC fractions from tubes to 96-well plates. NMR data were acquired on a Bruker Avance 400 spectrometer equipped with a Bruker 3 mm probe. NMR spectra were recorded using DMSO-d6 at 298 K, and the chemical shifts were referenced relative to the corresponding solvent signals (δ_H_ 2.50 for ^1^H-NMR and δ_C_ 39.51 for ^13^C-NMR). Mass spectrometric data were acquired on a Bruker APEX II 9.4T FTMS. UV spectra were recorded using the DAD on an Agilent 1100 HPLC system.

### Microbial Fermentation

All microorganisms were fermented in 500-mL flasks containing 100-mL selected production media. Actinomycetes were fermented at 28 °C at 200 rpm for 7 days while fungi were fermented at 22 °C at 200 rpm for 14 days.

### Extraction Process

Wet Diaion HP20 slurry (20 g) and wet Amberlite XAD-7 slurry (20 g) were added to liquid fermentation broth (400 mL). After shaking for 60 min, the pellet containing HP20, XAD-7 and cell mass was collected by either centrifugation or filtration. The collected pellet was washed with DI water (400 mL). The pellet was then extracted twice with MeOH (200 mL, total of 400 mL). One milliliter of each extract was sampled, concentrated to dryness, and resuspended in DMSO (100 μL) for LCMS-DAD-ELSD analysis and antimicrobial testing. The remaining crude MeOH extract was concentrated by rotary evaporation and then divided into two equal portions, one part for fractionation and one portion for long-term storage as reference material.

### Preparative HPLC Separation

Crude extracts for fractionation were dissolved into DMSO-MeOH-H_2_O (5 mL) and filtered using Whatman Autovial^®^ 0.45 μm glass microfiber filters. The extracts were separated into 10 fractions using the solvent gradient system outlined below in Table 1.

**Table 1:** Gradient solvent system used to generate the pre-fractionated library by preparative RP-HPLC.

Time	Flow Rate	H_2_O	MeOH	Collector
(min)	(ml/min)	(0.005% TFA)	(0.005% TFA)	
0	5	73	27	
3	30	73	27	
6	30	35	65	
8	30			Start
10	30	0	100	
15	30	0	100	
16	30	73	27	
17	30	73	27	
18	30	5	95	Stop
23	30	0	100	
28	30	0	100	
29	30	73	27	
34	30	73	27	
35	5	73	27	

The fractions were dried in the Thermo Savant SpeedVac systems and then resuspended in DMSO (1.8 mL) for plating into 96-well plates.

### LC-MS-ELSD Analysis

LCMS-ELSD data were acquired using an Agilent 1100 equipped with a diode array detector, LC/MSD SL using an ESI source, and a Sedere SEDEX 75 evaporative light scattering detector. Samples were analyzed using reversed-phase HPLC (Agilent Eclipse XDB-C18, 3.5 μm, 3.0 x 75 mm) with a solvent gradient of 5% A in B to 95% A in B over 10 min, holding at 95% A in B for 2 min (A = CH_3_CN plus 0.025% formic acid; B = H_2_O plus 0.025% formic acid; flow rate = 0.8 mL/min).

### Pure Compounds

Compound 70254-F08 [Equisetin (**2**) or Paecilosetin (**3**)]. UV (MeCN/H_2_O) λ_max_ 206, 234, 300 nm; HRESIMS *m/z* 374.223236 [M + H]^+^ (calcd for C_22_H_32_NO_4_, 374.23258).

Compound 70360-F09 [Trichloronorlichexanthone (**4**)]. UV (MeCN/H_2_O) λ_max_ 214, 254, 322 nm; HRESIMS *m/z* 358.92850 [M – H]^-^ (calcd for C_14_H_13_BrClO_4_, 358.92863).

Compound 70103-B09 [Preussomerin G (**6**)]. UV (MeCN/H_2_O) λ_max_ 222, 270, 348 nm; HRMS *m/z* 363.05083 [M – H]^-^ (calcd for C_20_H_12_O_7_, 363.05103).
